# Wild Animal Mortality Monitoring and Human Ebola Outbreaks, Gabon and Republic of Congo, 2001–2003

**DOI:** 10.3201/eid1102.040533

**Published:** 2005-02

**Authors:** Pierre Rouquet, Jean-Marc Froment, Magdalena Bermejo, Annelisa Kilbourn, William Karesh, Patricia Reed, Brice Kumulungui, Philippe Yaba, André Délicat, Pierre E. Rollin, Eric M. Leroy

**Affiliations:** * Centre International de Recherches Médicales de Franceville, Franceville, Gabon;; † European Union Project Cybertracker Monitoring Programme, Libreville, Gabon;; ‡ Universidad de Barcelona, Barcelona, Spain;; § Wildlife Conservation Society, Bronx, New York, USA;; ¶ Centers for Disease Control and Prevention, Atlanta, Georgia, USA;; # Institut de Recherche pour le Développement, Franceville, Gabon

**Keywords:** Ebola virus, wildlife, great apes, Duiker, outbreak, Gabon, Congo, network, monitoring, prevention, research

## Abstract

All human Ebola virus outbreaks during 2001–2003 in the forest zone between Gabon and Republic of Congo resulted from handling infected wild animal carcasses. After the first outbreak, we created an Animal Mortality Monitoring Network in collaboration with the Gabonese and Congolese Ministries of Forestry and Environment and wildlife organizations (Wildlife Conservation Society and Programme de Conservation et Utilisation Rationnelle des Ecosystèmes Forestiers en Afrique Centrale) to predict and possibly prevent human Ebola outbreaks. Since August 2001, 98 wild animal carcasses have been recovered by the network, including 65 great apes. Analysis of 21 carcasses found that 10 gorillas, 3 chimpanzees, and 1 duiker tested positive for Ebola virus. Wild animal outbreaks began before each of the 5 human Ebola outbreaks. Twice we alerted the health authorities to an imminent risk for human outbreaks, weeks before they occurred.

## Abstract 2

An animal mortality monitoring network in Gabon and the Republic of Congo has demonstrated potential to predict and possibly prevent human Ebola outbreaks.

Ebola virus, a member of the *Filoviridae* family, causes severe hemorrhagic fever in humans and nonhuman primates. The human case-fatality rate ranged from 50% to 89%, according to the viral subtype, from the first outbreaks in Zaire and Sudan in 1976 to the 2003 outbreaks in the Republic of Congo (*1**–**4*). No effective therapy or prophylaxis exists, and Ebola is a major public health concern. The first recorded human Ebola outbreaks (Yambuku, Zaire, 1976; Nzara, Sudan, 1976 and 1979; Tandala, Zaire, 1977) occurred abruptly, from an unidentified source, with subsequent person-to-person spread (*1**,**2**,**5**,**6*). No trace of the virus was initially found in wild animals close to the outbreaks (*7**–**9*). In 1989, for the first time, a nonhuman primate outbreak due to a new subtype of Ebola virus, Ebola subtype Reston, occurred in a colony of *Macaca fascicularis* in a quarantine facility in Reston, Virginia, USA, after the introduction of monkeys from the Philippines (*10*). Ebola Reston caused severe hemorrhagic fever in monkeys, but no clinical cases of human infection were identified, even though anti-filovirus antibodies were found in quarantine facility personnel (*11*). Later, in 1994, Ebola-specific immunohistochemical staining was positive on necropsy specimens from 1 of 12 chimpanzees that died in the Tai forest of Côte d'Ivoire (*12*). During this outbreak, an ethnologist was infected while performing an autopsy on a chimpanzee carcass; this was the first documented case of human infection transmitted by a nonhuman primate (*13*). During the 1996 outbreak in Mayibout (Gabon), an epidemiologic survey showed that the index case-patients had been infected by contact with a chimpanzee carcass. Concurrently, many nonhuman primate carcasses were reported in the area close to the outbreak, but none was recovered (*14**,**15*). Recently, we showed that all the human Ebola virus outbreaks that occurred in the past 3 years in Gabon and the Republic of Congo resulted from multiple introductions of the virus from different infected animal carcasses (*16*). We describe the development, testing, and evaluation of an Animal Mortality Monitoring Network (AMMN) in northeastern Gabon and northwestern Republic of Congo designed to alert human and animal health authorities on emerging epidemics.

## Materials and Methods

### Epidemiologic Surveillance Network

**Figure 1:**
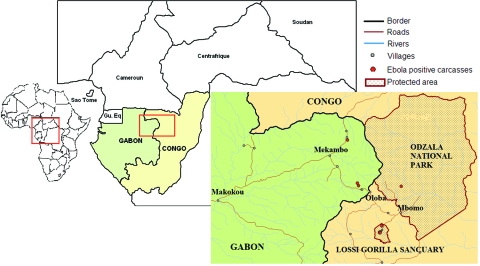
Map of the forest zone straddling the border between Gabon and Republic of Congo, showing (red points) the location of Ebola virus–positive carcasses, confirmed by testing in the Centre International de Recherches Médicales de Franceville biosafety level 4 unit during the 2001–2003 outbreaks in Gabon and Republic of Congo.

An alert network was set up by the Ministries of Health in hospitals and clinics in the different regions of Gabon and Republic of Congo, designed to report all human cases of viral hemorrhagic syndromes. Particular attention was paid to the northeastern region of Gabon, which had already been affected by outbreaks, and to its border region with Republic of Congo. Wildlife organizations such as the Wildlife Conservation Society (WCS), Programme de Conservation et Utilisation Rationnelle des Ecosystèmes Forestiers en Afrique Centrale (ECOFAC), and the World Wildlife Fund (WWF) were chosen to form the backbone of AMMN, in close collaboration with the Ministries of Forestry and Environment of the 2 countries. WWF was present in the Minkébé Reserve in Gabon, while ECOFAC was in charge of the Odzala National Park and the Lossi gorilla sanctuary in Republic of Congo (Figure 1).

All information on human cases of viral hemorrhagic syndrome or on the presence of dead animals in affected areas was centralized by a Viral Hemorrhagic Fever Committee (VHFC), composed of representatives of the Ministries of Health, Forestry, and Environment, the World Health Organization (WHO), wildlife agencies, and the Centre International de Recherches Médicales de Franceville (CIRMF). VHFC was also charged with sending specialized CIRMF teams to sample animal carcasses for diagnostic purposes. CIRMF is the regional reference laboratory for viral hemorrhagic fevers, and communicates its results to the Ministries of Health, Forestry, and Environment and to WHO.

### Ebola Outbreak Investigation: Human Case Data

The Gabonese and Congolese Ministries of Health, in close collaboration with WHO and its partners in the Global Outbreak Alert and Response Network (GOARN), were in charge of human epidemiologic investigations. A case of Ebola hemorrhagic fever was defined as any probable or laboratory-confirmed case, based on internationally recognized criteria (definition available from http://www.who.int/emc/diseases/ebola/ebola7.html).

### Ebola Outbreak Investigation: Animal Data

#### Collection Sites

From August 2001 to June 2003, carcasses were found on both sides of the Gabon–Republic of Congo border in the Ogooué Ivindo (Gabon) and West Basin (Congo) provinces (Figure 1). This entire area is covered by a *Marantaceae* and *Zingiberaceae* forest, with both open and closed canopies. The climate is equatorial, with 2 dry seasons (December–February and June–August) and 2 wet seasons (March–May and September–November). Mean rainfall is 1,500 mm per year and mean temperature is 24°C. Relative humidity always exceeds 80% (village of Mboko, Republic of Congo, 1995) (17).

#### Fauna

The large-animal fauna includes *Loxodonta africana* (Elephant), *Syncerus caffer* (Buffalo), *Tragelaphus* sp. (Sitatunga), *Cephalophus* sp. (Duiker), *Hylochoerus meinertzhagim* (Giant Forest Hog), *Potamochoerus porcus* (Red River Hog), *Gorilla gorilla*, *Pan troglodytes* (Chimpanzee), *Cercopithecus* sp. (Guenon), *Cercocebus* sp. (Mangabey), *Colobus* sp., *Panthera pardus* (Leopard), *Nandinia* (Two-spotted Palm Civet), *Civettidis civetta* (African Civet), *Genetta servalina* (Genet), mongoose sp., *Orycteropus afer* (Antbear), *Manis sp*. (Pangolin), *Atherurus africanus*, *Thryonomys swinderianus*, and *Python sebae* (*17**,**18*).

#### Carcass Detection

Local hunters (primarily adult and adolescent men of the Bakota, Bakola, Mboko, Mongom, and Pygmy tribes) were the main sources of information regarding the location of carcasses. Their reported sightings were confirmed by ECOFAC monitoring teams who recorded both the global positioning system (GPS) position on a CyberTraker field computer (available from http://www.cybertracker.co.za/) and carcass status before alerting VHFC.

#### Sampling Team and Methods

When wild animal carcasses were found, VHFC asked CIRMF to send a team to the site for diagnostic purposes. Sampling permits were granted by the Gabonese and Congolese Ministries of Forestry and Environment and Health. Owing to the isolated nature of the outbreak zone and its distance from CIRMF, a base camp was established nearby. GPS location of the carcasses, and the information provided on their state of decomposition, allowed the autopsy team to sample only the freshest carcasses.

**Figure 2:**
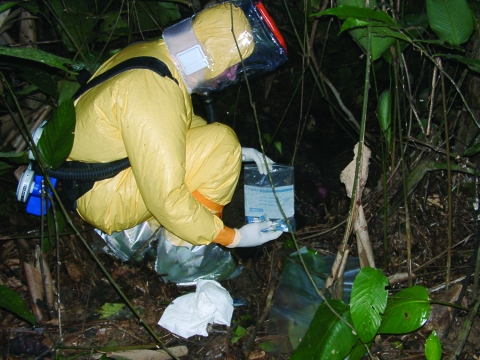
Field watertight clothes equipped with air filtration equipment, used for high-risk wild animal necropsy. Odzala National Park Republic of Congo, June 2003. Photo: P. Rouquet.

Ideally, the carcass sampling teams comprised a minimum of 5 persons (3 porters and 2 persons to perform the autopsy). One of the porters was charged with disinfection procedures. Digital photographs were taken. Necropsy was performed with high-level precautions, including watertight clothes Pro-Tech "C" (Tyvek, Contern, Luxembourg) equipped with air filtration equipment and Proflow Automask Litehood face shields (Delta Protection, Lyon, France) (Figure 2), and disposable lancets and forceps. A 2% chlorine spray was used to disinfect reusable equipment (masks and filtration apparatus), as well as the autopsy site and carcass remnants. Hermetic 60-L containers equipped with safety tops were used to transport reusable equipment and waste. Waste was returned to the main camp for incineration.

The nature of the samples taken depended on the state of the carcasses. When the carcasses were in good condition, 0.5-cm^3^ specimens of liver, spleen, muscle, and skin were taken. Half of the samples were placed in Nunc CryoTube vials (Nalge International, Rochester, New York, USA), which were placed in a small liquid nitrogen dry-shipper container (5.4 L) for cryopreservation (–196°C). The other samples were placed in Nunc CryoTube vials containing 10% formalin, for immunohistochemical testing. Bones were placed in hermetic containers. At the main camp, the dry-shipper contents were transferred into larger dry-shipper containers (20.3 L), which were then forwarded to the CIRMF laboratory at the end of the mission.

## Laboratory Studies

### Sample Preparation

Potentially infected specimens were collected and manipulated according to WHO guidelines on viral hemorrhagic fever agents in Africa (*19*). Muscle and skin tissue were fragmented and homogenized in phosphate-buffered saline, and the final supernatant was filtered for antigen detection and RNA amplification. Bones were cut, and internal tissue was scraped. Bone marrow or internal bone tissue was prepared in the same way as muscle and skin.

#### Testing

Muscle and skin tissue samples were tested by polymerase chain reaction (PCR), antigen detection, and, in some cases, immunohistochemical staining. Bone marrow and internal bone tissue were tested by PCR only.

#### Antigen Detection

Samples were used for antigen detection as previously described (*20*). Briefly, Maxisorp (Nalge International) plates were coated with a cocktail of 7 monoclonal antibodies against Ebola virus Zaire antigens; control plates were coated with normal mouse ascitic fluid produced from a parent myeloma cell line. Sample extracts (see above) were then added to the wells, followed by hyperimmune rabbit Ebola polyvalent antiserum and then peroxidase-conjugated goat antibodies against rabbit immunoglobulin G (IgG). The TMB detector system (Dynex Technologies, Issy-les-Moulineaux, France) was used to measure optical density.

#### DNA Amplification

For the detection of viral mRNA, total RNA was isolated from sample extracts by using the RNeasy kit (Qiagen, Hilden, Germany), and cDNA was synthesized from mRNA as previously described (*21*). Two pairs of degenerate primers corresponding to the L-gene of Ebola virus were used for 2 rounds of amplification, yielding a 298-bp fragment (5´-TATMGRAATTTTTCYTTYTCATT-3´ and 5´-ATGTGGTGGGYTATAAWARTCACTRACAT-3´ for primary PCR; 5´-GCWAAAGCMTTYCCWAGYAAYATGATGG-3´ and 5´-ATAAWARTCACTRACATGCATATAACA-3´ for nested PCR).

#### Immunohistochemical Staining

Formalin-fixed specimens were sent to the Centers for Disease Control and Prevention (Atlanta, Georgia, USA) for immunohistochemical staining as previously described (*22*).

## Results

### Human Outbreaks

From October 2001 to December 2003, 5 human Ebola virus outbreaks of the Zaire subtype occurred in the area straddling the border between Gabon (northeast) and Republic of Congo (northwest), with 313 cases and 264 deaths (*23**,**24*). The first outbreak occurred from October 2001 to May 2002, with a total of 92 cases and 70 deaths in Gabon and Republic of Congo. Epidemiologic investigations showed that at least 2 duikers, 2 chimpanzees, and 2 gorilla carcasses were involved or suspected of being involved in the infection of 6 human index patients. A second human outbreak began in January 2002 and ended in June 2002 in Entsiami Republic of Congo, with a total of 30 cases and 25 deaths. One gorilla and 1 duiker were suspected of involvement in 2 human index cases. A third outbreak occurred from May to June 2002 in Oloba Republic of Congo, with 13 cases and 12 deaths. A chimpanzee was shown to have infected the human index patient. The fourth outbreak occurred from December 2002 to April 2003 in Mbomo and Kéllé, Republic of Congo, with 143 cases and 128 deaths. Gorillas and duikers were suspected of infecting 3 human index patients. The last outbreak occurred from November 2003 to December 2003 in Mbanza and Mbomo, Republic of Congo, with 35 cases and 29 deaths. The source of infection of the human index patient was not clearly identified.

### Carcasses

**Figure 3:**
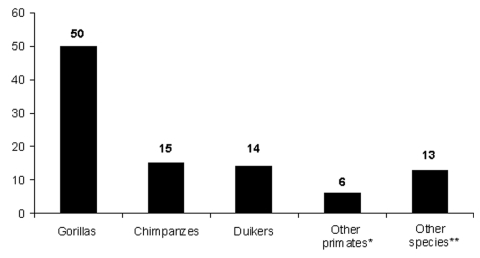
Species distribution of carcasses found in the forest straddling the border between Gabon and Republic of Congo (2001–2003). * = other primates: *Cercopithecus* sp.; †= other species: *Atherurus africanus* (1), *Genetta* sp. (3), *Loxodonta africana* (1), *Manis* sp. (1), *Mongoose* sp. (1), *Thryonomys swinderianus* (2), *Tragelaphus* sp. (1), *Python sebae* (2), and bird of prey (1).

**Figure 4:**
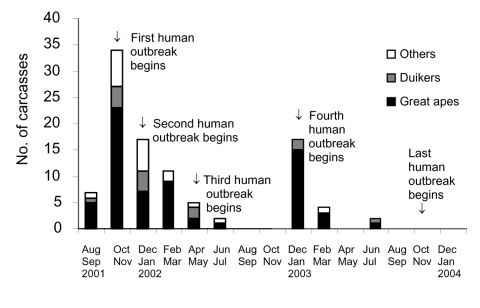
Temporal distribution of carcasses found in the forest straddling the border between Gabon and the Republic of Congo (2001–2003). Two peaks of mortality were observed: the first occurred in the Ekata region (Gabon) from November to December 2001 and the second from December 2002 to February 2003 in the Lossi gorilla sanctuary (Republic of Congo).

From August 2001 to June 2003, a total of 98 animal carcasses were found in an area of about 20,000 km^2^ (Figure 3). Carcasses of 3 principal species were recovered: 65 great apes (50 gorillas and 15 chimpanzees) and 14 duikers (Figure 3). Only 6% of carcasses sampled were in good condition (entire body); 57% were in poor condition (partial carcasses with muscles or skin); and 38% were in bad condition (bones only). Two peaks of animal deaths were observed (Figure 4). The first occurred in the Ekata region (Gabon) from November to December 2001, with 51 carcasses, including 30 great apes and 8 duikers. The second occurred from December 2002 to February 2003 in the Lossi gorilla sanctuary (Republic of Congo), with 20 carcasses, including 17 great apes, 2 duikers, and 1 *Cercopithecus cephus*.

### Laboratory Findings

An animal carcass was considered infected by Ebola virus if >1 of the 3 laboratory tests (antigen detection, DNA amplification, and immunohistochemical staining) was positive. When possible, DNA amplification was confirmed by sequencing the PCR products. Twenty-one gorilla, chimpanzee, and duiker carcasses were sampled in the wild and analyzed in the CIRMF biosafety level 4 (BSL-4) laboratory. Fourteen of these carcasses tested positive for Ebola virus, 6 in 2 or 3 tests and 8 in only 1 test (Table). Eight positive samples were muscles, and 6 were bones or bone marrow. All the muscle and skin tissue samples were tested by both PCR and antigen detection. In total, 10 gorillas, 3 chimpanzees, and 1 duiker tested positive. All the relatively well-preserved gorilla and chimpanzee carcasses tested positive. In contrast, well-preserved samples taken from carcasses of *C. cephus*, *Genetta* sp., and *Tragelaphus* sp. were negative.

**Table:** Results of laboratory analysis of animal carcasses found in forest between Gabon and the Republic of Congo, November 2001–June 2003*

Animal	Location								
	Area	GPS		Date	Tissue	Death	PCR	Ag	IHC
Ebola+ by 2 or 3 tests									
Gorilla	Zadié	0,7055N	14,2747E	Nov 2001	Muscle§	5 d	+	+	+
Gorilla	Lossi	0,2395N	14,4938E	Dec 2002	Muscle§	8 d	+	+	+
Gorilla	Lossi	0,2354N	14,4839E	Dec 2002	Muscle§	8 d	+	+	+
Gorilla	Mbanza	0,6987N	14,7029E	Jun 2003	Muscle§	5 d	+	+	N/A
Chimp†	Lossi	0,2387N	14,4885E	Dec 2002	Muscle§	3 d	–	+	+
Chimp	Lossi			Feb 2003	Muscle¶	10 d	+	+	N/A
Ebola+ by 1 test									
Gorilla	Zadié	1,1669N	14,1650E	Feb 2002	Bone marrow#	1 mo	+	N/A	N/A
Gorilla†‡	Zadié	0,7310N	14,2644E	Mar 2002	Bone#	3 wk	+	N/A	N/A
Gorilla†‡	Zadié	0,7310N	14,2644E	Mar 2002	Bone#	3 wk	+	N/A	N/A
Gorilla	Lossi	0,2348N	14,4852E	Dec 2002	Bone#	2 wk	+	N/A	N/A
Gorilla	Lossi	0,2346N	14,4823E	Dec 2002	Bone#	2 wk	+	N/A	N/A
Gorilla	Lossi	0,2987N	14,5075E	Feb 2003	Muscle¶	8 d	–	+	N/A
Duiker	Lossi	0,2293N	14,4892E	Dec 2002	Bone#	2 wk	+	N/A	N/A
Chimp†	Lossi	0,2387N	14,4885E	Dec 2002	Muscle¶	12 h	–	+	–
Tested and Ebola–									
Gorilla‡	Zadié	0,6510N	14,2375E	Mar 2002	Skull#	1 mo	–	N/A	N/A
Duiker	Lossi	0,2376N	14,4882E	Dec 2002	Bone#	3 wk	–	N/A	N/A
Duiker	Lossi			Jun 2003	Skin§	2 d	–	–	N/A
*Cercopithecus cephus*	Lossi	0,2737N	14,5163E	Feb 2003	Muscle§	3 d	–	–	N/A
Genet	Zadié	0,6749N	13,8851E	Nov 2001	Muscle¶	5 d	–	–	N/A
Genet	Zadié	0,6771N	14,2937E	Feb 2002	Muscle§	2 d	–	–	N/A
Sitatunga	Zadié	0,9560N	13,7776E	Apr 2002	Muscle§	3 d	–	–	N/A

*GPS, global positioning system (CyberTracker field computer); PCR, polymerase chain reaction; Ag, antigen detection; IHC, immunohistochemical tests; N/A, not applicable. †Mother and infant. ‡1-month delay between the field and the laboratory and preserved in bad conditions. §Sample found in good condition. ¶Sample found in poor condition. #Sample found in very poor condition (bone only).

## Discussion

We describe the successful implementation of a surveillance network of Ebola outbreaks in wild large mammals. We often identified wild animal outbreaks before human Ebola outbreaks. Twice this enabled us to alert the health authorities of Republic of Congo and Gabon to an imminent risk for human outbreaks, after the discovery of carcasses of Ebola virus–infected animals.

Human Ebola outbreaks in this region have always occurred in remote areas, raising major logistic problems. Roads are often barely passable, and means of communication are frequently nonexistent. The carcass detection and investigation network therefore had to rely on teams already present in these forest zones, and notably those possessing radios or satellite telephones. Conservation organizations such as ECOFAC, WCS, and WWF were thus the ideal partners. ECOFAC monitoring teams played a critical role by exploring remote forest zones, capitalizing on the information provided by villagers and hunters.

**Figure 5:**
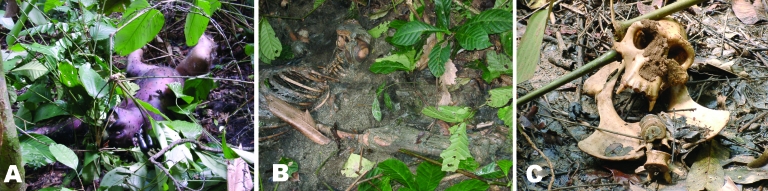
State of the wild animal carcasses found in the field, Lossi gorilla sanctuary, Republic of Congo, December 2002. Carcasses decompose very rapidly in the equatorial forest. Photo: P. Rouquet. A) Female chimpanzee, 3 days after death. B) Female gorilla, 7 days after death. C) Female gorilla, 21 days after death.

Performing an autopsy on high-risk animal carcasses requires heavy equipment, highly qualified personnel, and experienced veterinarians, as illustrated by the case of the Swiss anthropologist who was infected after examining a chimpanzee carcass without adequate protective measures in the Tai forest (*13*). Carcasses decompose very rapidly in the equatorial forest: an adult male gorilla carcass (≈150 kg) takes only 10 days to decompose entirely, i.e., be reduced to a heap of bones and hair (Figure 5). Carcasses observed 3–4 days after death bear few signs of scavenger activity but are covered with fly eggs and maggots. Maggots consume the entire flesh within 5 to 10 days, while scavengers (mainly mongoose) take pieces and disseminate them around the site. Thus, after ≈3weeks, only a few bones bearing small-mammal gnaw marks remain.

Although the PCR technique used by CIRMF can detect Ebola virus genetic material in carcasses 3–4 weeks old, the material is often degraded and incomplete. Often, only a small sequence of the L-gene (RNA polymerase) can be analyzed, and this cannot be used for strain identification. Furthermore, degraded samples increase the false-negative rate (*25*). Rapid sampling is therefore crucial for successful diagnosis, and the availability of a small aeroplane was particularly helpful in certain cases. The presence of the CIRMF BSL4 laboratory relatively close to the outbreak area was a considerable advantage.

Using a combination of 3 laboratory techniques (PCR, immunohistochemical staining, and antigen capture), we showed for the first time that wild gorillas and chimpanzees can be decimated by Ebola. Bones of a *Cephalophus dorsalis* carcass also tested positive for Ebola virus by reverse transcription (RT)-PCR, indicating that a third wild species may be naturally susceptible. In Africa, only chimpanzees had previously been diagnosed as positive for Ebola virus, by immunohistochemical testing, in the Tai forest of Côte d'Ivoire, and were considered the cause of the human outbreak in Mayibout (Gabon) (*12**,**14**,**15*). The large number of carcasses found in this region, together with the results of animal population censuses conducted in the Lossi reserve before and after outbreaks, indicates that great apes are affected massively and duikers to a lesser extent (*16**,**26*). The lowland gorilla population density in this region (<6 times as high as the chimpanzee population density) is among the highest in the world (<10 gorillas/km^2^) (*27*), which likely explains why more gorilla carcasses than chimpanzee carcasses were found. High population density can amplify outbreaks but cannot alone explain their severity. Small monkeys, although abundant in this area, do not seem to be affected. Only 1 carcass of *Cercopithecus cephus* was found; it was in good condition but was negative by RT-PCR and antigen capture (Table). Some *Potamochoerus* *porcus* carcasses were reported by hunters but none could be sampled. Carcasses of large animals are more likely to be found than those of small animals, because the time taken for a carcass to decompose depends on its size.

The source of gorilla infection is unknown, but several lines of evidence point to direct infection by >1 natural hosts. First, the detection of different strains of Ebola virus in gorilla carcasses located only a few kilometers apart argues against a major role of gorilla-to-gorilla transmission. Indeed, Ebola virus remains genetically stable during a given outbreak, from the first to the last case (*28**,**29*), whereas we obtained 4 different glycoprotein sequences (E.M. Leroy, P. Rouquet, unpub. data) from samples of gorillas and chimps located in the Lossi sanctuary. The large distance separating positive carcasses found during a short period, and the existence of physical barriers such as roads and rivers, also supports direct transmission from a natural host. Finally, the occurrence of simultaneous outbreaks in 2 or 3 different species that display little interspecies contact (*30*) provides further evidence that gorillas and chimpanzees are directly infected by >1 natural hosts. However, cases of gorilla-to-gorilla transmission cannot be ruled out, especially within a given group. Indeed, 5 gorilla carcasses belonging to the same group were found in a close area in the Lossi sanctuary. Ebola outbreaks in gorilla groups may result in their rapid dissolution, especially if the dominant male is rapidly affected, which forces possibly infected females to integrate into another group. However, this type of intergroup transmission appears to be marginal.

Chimpanzees are probably infected by the same mechanisms as gorillas. During the Tai outbreak in Côte d'Ivoire, carnivorous behavior (especially consumption of *Colobus* monkeys) was the suspected source of infection (*12*), but this notion is challenged by the infection of gorillas, which are almost exclusively herbivorous. However, chimpanzees are considered to be the primate species whose behavior (mainly fighting, social grooming, sexual activities, and predation) carry the highest risk for both intra- and interspecies pathogen transmission (*30*). This idea is supported by the detection of the infected carcasses of a mother and her 1-year-old offspring. Repeated contact between young individuals and their mothers is known to be a significant risk factor for Ebola virus transmission (*2**,**6*).

Duikers represent a special case. Although they are the most common large-mammal species in this region, few carcasses were found. This circumstance may be due to the lack of interactions among individuals, as duikers generally live alone or in pairs. Some duikers, despite being herbivorous, eat the flesh of decomposing carcasses (K. Abernethy, unpub. data). Thus, in addition to being directly infected by the natural host(s), duikers might also become infected by licking or eating fresh carcasses of Ebola virus–infected animals. This scenario would play a marginal role, however, because carcasses are only infective for 3 or 4 days after the animal's death (E.M. Leroy, P. Rollin, unpub. data). Furthermore, we observed little scavenging of carcasses during the first days after the animal's death.

Serum from a survivor of the human outbreak in Mekambo (Grand Etoumbi, March 2002), who had direct contact with a gorilla carcass, was positive for Ebola virus–specific IgG. Ebola virus L gene sequences were detected in bone marrow samples of this gorilla, conclusively linking the 2 cases. Thus, the last outbreaks in Mekambo (Gabon, 2001) and Lossi (Republic of Congo, 2002–2003) confirm that wild animal mortality can reveal Ebola virus propagation in the forest ecosystem and indicate a role of wild animals as "vectors" in human outbreaks.

No effective medical treatment or vaccine exists for Ebola virus infection. The only way of minimizing human cases is to break the chain of human-human transmission. Humans do not seem to be at a major risk for infection by the unidentified natural host(s). Large outbreaks among wild animals can amplify human outbreaks by increasing the number of index transmission events. Therefore, reducing contacts between humans and dead wildlife can reduce the risks for transmission.

**Figure 6:**
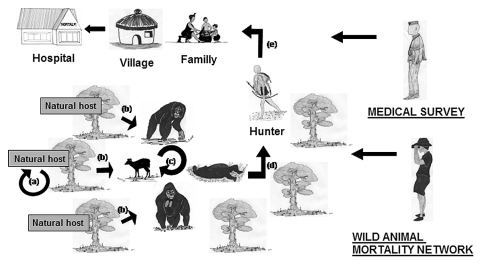
Schematic representation of the Ebola cycle in the equatorial forest and proposed strategy to avoid Ebola virus transmission to humans and its subsequent human-human propagation. Ebola virus replication in the natural host (a). Wild animal infection by the natural host(s) (b), no doubt the main source of infection. Wild animal infection by contact with live or dead wild animals (c). This scenario would play a marginal role. Infection of hunters by manipulation of infected wild animal carcasses or sick animals (d). Three animal species are known to be sensitive to Ebola virus and to act as sources of human outbreaks, gorillas, chimpanzees, and duikers. Person-to-person transmission from hunters to their family and then to hospital workers (e). The wild animal mortality surveillance network can predict and might prevent human outbreaks. Medical surveillance can prevent Ebola virus propagation in the human population.

Epidemiologic surveillance of animal mortality rates can thus help prevent the emergence of the disease in human populations (Figure 6). At the time of the Kéllé (Republic of Congo) outbreak, our network detected infected gorilla carcasses (Lossi, December 6, 2002) 3 weeks before the disease emerged in humans (December 25, 2002), showing active Ebola virus propagation in this area. We were thus able to warn health authorities of an imminent human outbreak in the region. Nonetheless, a human outbreak occurred. In June 2003, we issued a new alert on a risk for human outbreaks after the discovery of an infected gorilla carcass near the village of Mbanza (Republic of Congo). An outbreak occurred in this village in November 2003. These failures suggest that human and animal health authorities need to work together more closely. In the future, health authorities need to educate local populations on the risk for infection through contact with carcasses at all times. During expected disease outbreaks, health authorities need to be able to respond immediately by sending teams to affected areas (*24*). The early successes of the network in this area warrant its extension to all countries with known outbreaks of hemorrhagic fevers. The participation of new frontline partners, such as foresters, would be invaluable to expend logistical existing capacity provided largely by field conservationists. Finally, as the capacity of such a system to react rapidly is crucial for its success, sampling teams should be created to collect material and obtain virologic testing results with a minimum of delay in other countries harboring hemorrhagic viruses. An efficient animal mortality monitoring network backed up by a rapid reaction system would allow public health authorities to predict and possibly prevent human Ebola outbreaks.
